# Upper tibial MRI vascular marks lost in early knee osteoarthritis

**DOI:** 10.1186/s13018-018-0991-y

**Published:** 2018-11-12

**Authors:** Michael Beverly, Gil Stamm, Thomas W. Hamilton, David W Murray, Hemant G Pandit

**Affiliations:** 1 NDORMS, Uiversity of Oxford, Botnar Research Centre, Nuffield Orthopaedic Centre, Windmill Road, Headington, Oxford, OX3 7LD UK; 2 Leeds Institute of Rheumatic and Musculoskeletal Medicine, Chapel Allerton Hospital, Chapeltown Road, Leeds, LS7 4SA UK

**Keywords:** MRI, Subchondral, Vascular, Tibia, Kellgren-Lawrence, Osteoarthritis

## Abstract

**Background:**

We describe upper tibial radiating vascular marks on MRI scans. They are lost in early osteoarthritis (OA).

**Methods:**

A literature search revealed no previous description of upper tibial MRI radial vascular marks. Fifty-six consecutive patients with anteroposterior knee X-rays and an axial PD_SPAIR MRI scan of the same knee within 1 year were studied. Their mean age was 53.1 years (range 22–85) with 27 males and 29 females. The medial and lateral compartments of each knee were scored for osteoarthritis using the Kellgren-Lawrence (K-L) classification. Marks on the MRI scans were counted by layer and quadrant position.

**Results:**

Radial vascular marks were present in the first axial upper tibial subchondral slice, peaked between 6 and 10 mm depth and were absent by 16 mm depth. There was no association with age, left or right knee, BMI, or weight. There was more K-L graded OA medially and more vascular marks laterally. There was an inverse correlation between the number of marks and early grades of osteoarthritis medially (*p* < 0.001) and laterally (*p* < 0.002).

**Conclusion:**

We demonstrate previously undescribed subchondral vascular marks on axial MRI scans of the tibia and their inverse correlation with the presence and severity of early knee osteoarthritis. Our work offers a new insight into the possible vascular aetiology of osteoarthritis and potentially a means of earlier diagnosis and a therapeutic target.

## Background

Osteoarthritis (OA) of the knee causes significant economic costs to society and morbidity affecting much of the ageing population [1]. Diagnosis is traditionally by clinical and radiological examination. Only in the later stages of the disease do X-rays reveal loss of joint space and deformity.

The etiology of primary OA is usually thought to be multifactorial with age-related wear and tear, overload, trauma, genetic factors, and systemic disease being recognised as major contributors. Vascular disease has not usually been considered a significant factor [2].

To outline the arterial anatomy of the bone, Trueta used barium sulphate emulsion injection and X-rays [3]. This approach was improved by Brookes who cleared the calcified tissue with acid [4]. The mapping of bony venous drainage proved to be more challenging. Retrograde venous injection of the bone is difficult because there are usually several superficial veins draining a limb contrasting with one or two supplying arteries, venous valves prevent retrograde flow, and venous shunts may operate. Only a small percentage of limb blood flow is through the bone. As a result, previous researchers have often injected the arteries and looked at the sagittal or coronal plane anatomy. They then assumed that there was a reciprocal venous pattern such as is demonstrated in Grey’s Anatomy [5]. Crock described a subchondral plexus of vessels in vertebral bodies using Indian ink and decalcification but little interest has otherwise been directed at subchondral blood vessel patterns in weight bearing joints [6, 7].

Denham realised that forces of several times body weight were transmitted across joints during ordinary activity [8]. It is possible that weight bearing might raise subchondral intraosseous pressure [9, 10]. A raised intraosseous pressure has itself been associated with pain and osteoarthritis [11–14]. It is likely that if the subchondral region is subject to high forces or pressures during activity there could be anatomical or vascular modifications to cope [15].

**Fig. 1:**
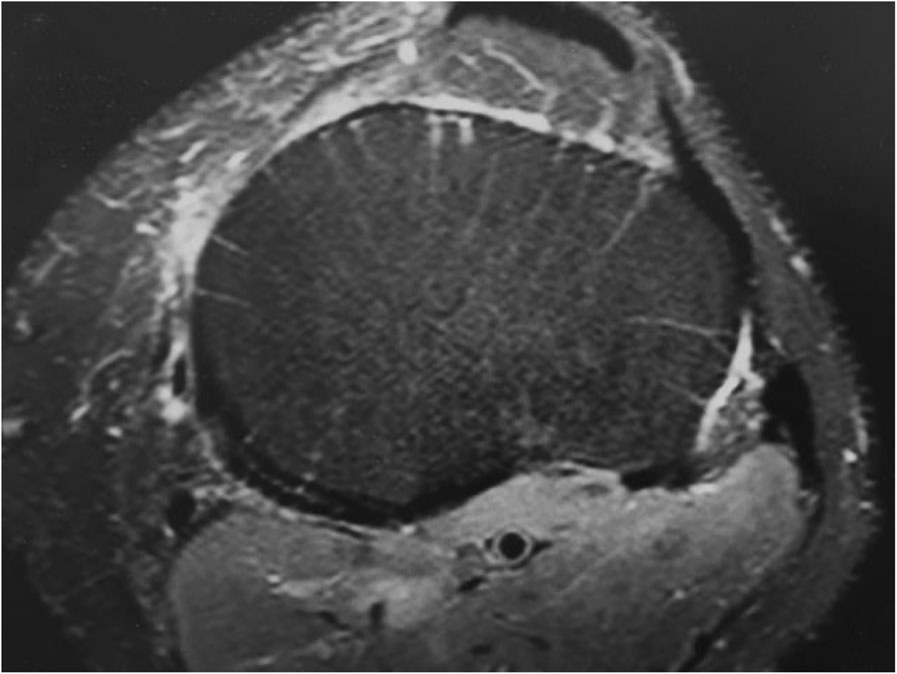
Example of upper tibial axial MRI slice. A typical axial upper tibial PD_SPAIR MRI slice at level 3. White radial vascular marks are evident

We observed axial subchondral radiating marks in the proximal tibia on (proton density spectral presaturation with attenuated inversion recovery) PD_SPAIR water bright or fat suppressed axial MRI scans of the knee as in Fig. 1. A literature search failed to provide any previous description. We found that the marks were present in axial scans of several joints but for the purpose of the present work could best be studied in axial MRI slices of the relatively flat upper tibia. In this study, we were interested in the general incidence and distribution of the marks and in their association with arthritis in patients referred to our knee clinic. We studied the distribution of these marks in terms of their distance below the tibial surface and the pattern of distribution within quadrants of the axial plane. We then explored their association with early osteoarthritis of the knee assessed by Kellgren-Lawrence (K-L) grade on contemporary plain X-rays.

## Methods

### Literature search

A literature search returned no relevant references or previous papers when using key words including MRI, tibia, subchondral, intraosseous, vessel, or vein.

### Analysis

This preliminary study had University and NHS ethical approval (OUH R&D Ref 12541, April 2017). We reviewed the anonymised images of patients who attended a knee clinic over a 6-month period in a University Teaching Hospital. All patients who had a plain standing anteroposterior X-ray of the knee and an MRI scan of the same knee within a 12 month period were included. There were 60 pairs of images initially. We were not aware of their diagnoses and did not exlcude inflammatory arthritis. There was no attempt to estimate the varus or valgus posture of the joint. Patients were only excluded if their X-ray or MRI scan showed previous significant knee surgery such as knee replacement or internal fixation for trauma, ligament repair or other significant pathology. There remained 56 patients, 27 male and 29 female patients. There were 28 left knees and 28 right knees. The patients ranged in age from 22 to 85 years old with a mean age of 53.1 years. The MRI sequences were from a standard knee protocol in a 3-Tesla machine used in routine clinical practice at our institute. The protocol included axial proton density spectral presaturation with attenuated inversion recovery (PD_SPAIR) images.

Two researchers, one an experienced orthopaedic surgeon and the other a medical student, assessed all the X-rays on a standard computer monitor. On the AP films, the medial and lateral compartments of the knee were independently scored for OA using the Kellgren-Lawrence classification [16]. The same researchers also assessed the MRI scans. They were blinded as to the K-L grade of the X-rays and as to each other’s findings. In addition, all X-rays and MRI scans were reassessed by both observers 3 weeks after the initial measurements in order to calculate inter- and intra-observer variability. The observers were blind as to their previous results.

### MRI scoring method

**Fig. 2:**
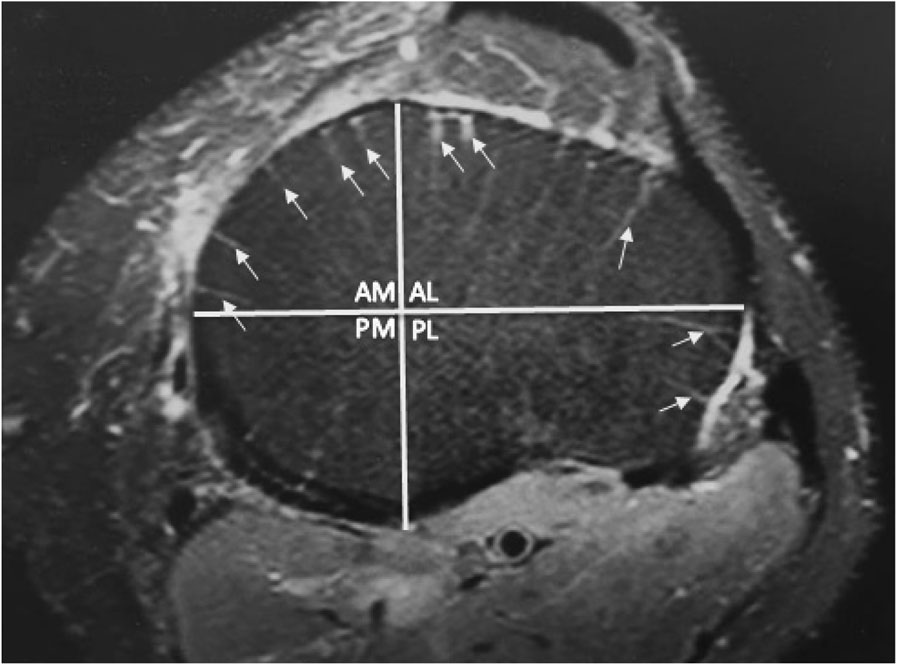
Example of quadrant division and scoring. The same left knee level 3 MRI slice seen in Fig. 1 with scoring marks. This slice scored 5, 0, 2, and 3 for anteromedial, posteromedial, posterolateral, and anterolateral quadrants

The MRI images were reviewed one slice per full screen on the same viewing monitor without changing the brightness, contrast, or magnification. The first tibial slice or layer was taken as the most proximal subchondral axial slice of the knee to show only the tibial bone. The scanning protocol was for slices 3.0 mm thick spaced 0.3 mm apart. The total scanned depth therefore amounted to 16.5 mm from the center of the first slice to the center of the sixth slice. The axial scans were divided into quadrants vertically and horizontally at their maximum diameter as in Fig. 2. The vascular marks were counted by layer and by quadrant. A vascular mark was defined as a radiating white line which reached the outside edge of the tibia. Vascular marks were not counted if they did not reach the outer edge of the tibia or if they were less than 5 mm long.

### Statistical methods

Inter- and intra-observer agreement for Kellgren-Lawrence grade and number of vascular marks on MRI was determined using a weighted kappa assessment for ordinal data. Inspection of the data revealed both Kellgren-Lawrence grade and number of vascular marks to be skewed and as such non-parametric analysis was performed. To assess the association between location based on analysis of four quadrants of the proximal tibia and the number of vascular marks observed, a Kruskal Wallis test was performed with Mann Whitney *U* post hoc testing to assess for differences between individual quadrants. Where post hoc tests were performed, a Bonferroni correction was applied to the significance level to account for multiple testing. To assess the association with age a Spearman rank test was performed with Mann Whitney *U* tests performed to assess for associations with gender and left and right knees. To assess for association with BMI and weight, Spearman rank tests were performed. To assess for an association with grade of osteoarthritis a Spearman rank test was performed. To assess for differences in vascular marks within grade of osteoarthritis, Mann Whitney *U* tests were performed.

All analyses were performed using SPSS Version 22 (IBM Corporation, Armonk, New York). Statistical significance was set at *p* < 0.05.

## Results

### Time between X-ray and MRI

The mean time interval between AP radiographs and MRI scans of the ipsilateral knee was 3.65 months (SD, 3.21; range, 0.03–11.87).

### Inter- and intra-observer reliability

There was close agreement between the graders, one an experienced consultant orthopaedic surgeon and the other a medical student. Inter and intra-observer reliability was moderate to excellent. Inter-observer agreement for Kellgren-Lawrence Grade and total number of vascular marks was 0.73 and 0.82 respectively. Intra-observer agreement for Kellgren-Lawrence Grade and total number of vascular marks was 0.89 and 0.93 respectively.

### Position below surface

**Fig. 3:**
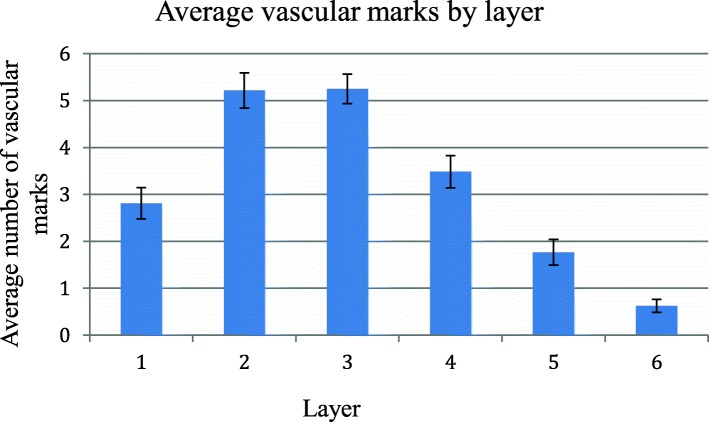
Incidence of vascular marks by layer below tibial surface. Vascular marks in the first six axial layers below the upper tibial articular surface. Scans slices were 3.0 mm thick and 0.3 mm apart giving a depth of 16.5 mm from the middle of slice 1 to the middle of slice 6, *n* = 56 knees, error bars SEM

Vascular marks appeared in the first subchondral tibial layer, increased in number in the second and third layers, and virtually disappeared by the sixth as in Fig. 3.

### Distribution

**Fig. 4:**
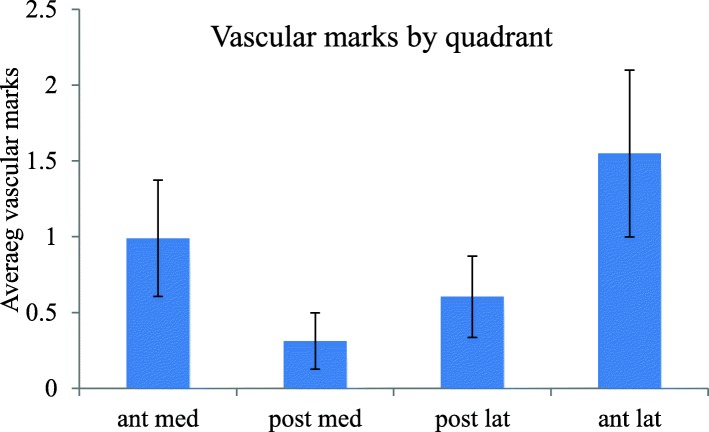
Incidence of vascular marks by quadrant. The distribution of vascular marks between the quadrants showed more marks anterolaterally and fewer posteromedially, *n* = 56 knees, error bars SEM

The distribution between the quadrants was analysed. The distribution of vascular marks was found to differ significantly between different quadrants (*p* < 0.001) with the anterolateral quadrant containing the most vascular marks and the posteromedial quadrant the fewest. Post hoc testing revealed a significantly higher number of vascular marks anteriorly compared to posteriorly in both the medial (*p* < 0.001) and lateral compartments (*p* < 0.001) and a significantly higher number of vascular marks laterally compared with medially across both the anterior (*p* < 0.001) and posterior quadrants (*p* < 0.001) as in Fig. 4.

### Age and left and right knees

No association between age and total number of vascular marks was detected (*p* = 0.30). No association between the left and right knees and total number of vascular markings was detected (*p* = 0.10).

### BMI and weight

There was no association between the total number of vascular markings and patient’s BMI (*p* = 0.50) or their body weight (*p =* 0.87).

### Gender

Fewer vascular marks were seen in females compared to males (*p* < 0.001). Females had more osteoarthritis with a mean K-L score for females of 1.83 medially (SD 0.83) and 1.43 laterally (SD 0.73). The mean K-L score for males was 1.54 medially (SD 0.78) and 1.20 laterally (SD 0.40).

### Pattern of osteoarthritis

The plain X-ray Kellgren-Lawrence distribution of osteoarthritis grades between the knees was as in Table 1. There was more osteoarthritis medially than laterally.

**Table 1:** Incidence of K-L grade medial and lateral sides

K-L grade in 56 knees	Numbers of patients at each K-L grade medial	Numbers of patients at each K-L grade lateral
0	16	32
1	20	18
2	15	4
3	3	2
4	2	0
Mean K-L score	1.2	0.57

Distribution of patients at each K-L grade. There was proportionately more osteoarthritis medially, *n* = 56 for both sides

### Correlation with OA

**Fig. 5:**
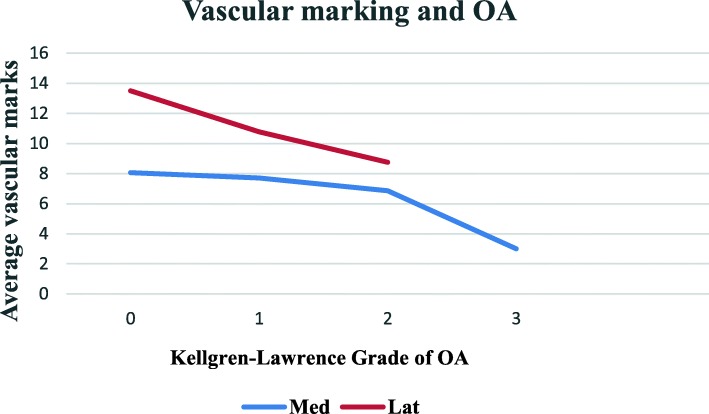
Loss of vascular marks with increasing grade of OA. The reduction in vascular mark numbers with increasing K-L grade for medial and lateral sides

Analysis showed a negative correlation between the grade of osteoarthritis and the number of tibial vascular marks in the medial (Spearman rho − 0.31, *p* < 0.001) and the lateral (Spearman rho − 0.29, *p* < 0.002) compartments of the knee. There were differences in vascular marks within the OA grades between medial and lateral. Mann Whitney *U* tests at each K-L grade showed significant differences between the two compartments in grade 0 (*p* < 0.001) and 1 (*p* < 0.005) but no significant difference at K-L grade 2 (*p* = 0.271) as in Fig. 5. There were insufficient numbers for analysis of grades 3 and 4 as in Fig. 5.

**Fig. 6:**
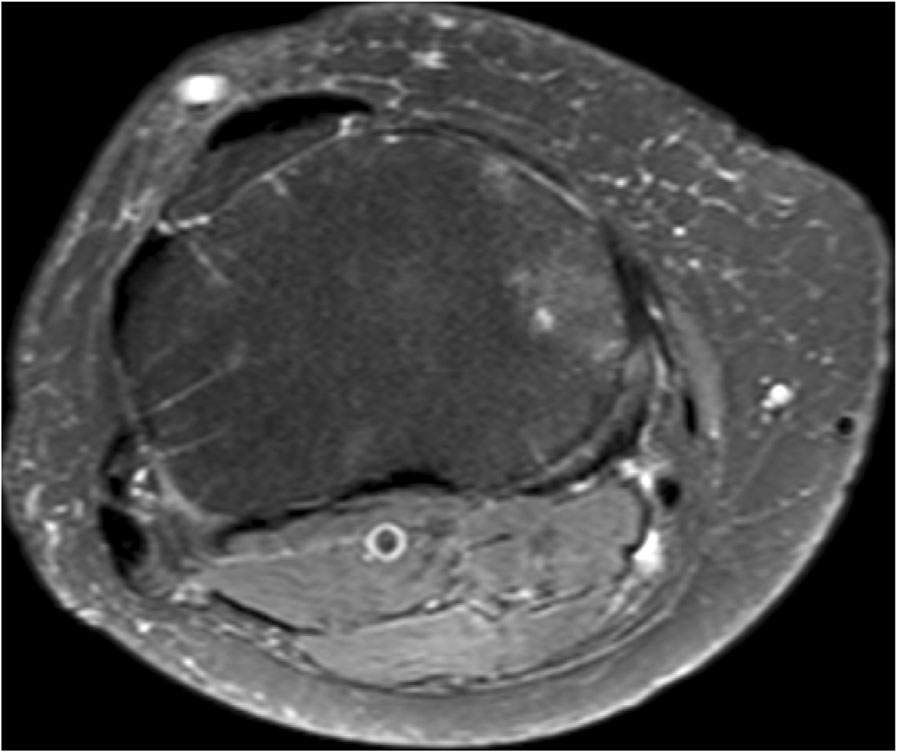
Axial MRI slice with medial OA. A typical slice at level 3, right knee, with K-L grade 2 medial osteoarthritis on plain x-ray. The medial vascular marks are absent while the K-L grade 0 lateral side retains the vascular marks

As the number of vascular marks declined, the grade of osteoarthritis increased. A typical example is seen in Fig. 6.

## Discussion

We have identified previously undescribed radial vascular markings on MRI scans of the upper tibia. The white lines are also visible on T2 or water bright MRI scans but he marks are best seen in axial T2 or PD_SPAIR scans. Radiological opinion was that these marks are vascular, and probably venous. Reasons given include that they are longitudinal channels, water enhancing, occasionally in tributary form, increase in size as they reach the periphery, penetrate the cortex, and appear to link with periosteal vessels. There is a slow flow rate in these vascular marks during MRI scanning in the recumbent patient. Arteries are generally narrower, and the higher flow rate makes their contents less visible. In the popliteal artery in Figs. 1 and 6, the contents of the artery are dark whereas the superficial vein contents are bright. A literature search failed to find any relevant papers that described subchondral vessels or veins or that addresses the loss of subchondral vessels associated with osteoarthritis [17–19].

We chose the Kellgren-Lawrence method to grade osteoarthritis of the knee on plain AP X-rays because of its simplicity and our access to the corresponding plain X-rays. The majority of patients had osteoarthritis rankings in the lower K-L grades from 0 to 2. In our practice, few patients with severe osteoarthritis would be sent for an MRI scan. We acknowledge that the K-L grading system is a relatively insensitive tool for separating early grades of OA. Many patients with grade 0 or 1 osteoarthritis on plain radiographs have well-established cartilage disease on arthroscopy which is not yet apparent on X-rays [20].

The medial side is the more frequent site for primary knee OA and patients presenting in our clinic generally had more early medial osteoarthritis. The K-L grading system cannot easily differentiate between early grades of OA. The loss of vascular marks on MRI may be a means of detecting early OA. In this study of a group with varying degrees of early osteoarthritis, there were fewer vascular marks on the medial side generally and this was associated with more advanced K-L scores. We note that females usually have smaller joints than males. We acknowledge that this may be the reason for them having fewer vascular marks. However, we found that females had fewer marks and higher mean K-L scores for osteoarthritis both medially and laterally.

MRI image quality depends on many variables [21]. The MRI scanning protocol was that used routinely in our department on a 3-Tesla machine. For analysis, we used a standard PC screen such as those found on any ward based PACS system to view the images. We acknowledge that up to 75% of the data is lost in those images compared with the specialised screens available in an X-ray department.

There are other limitations to our study. The study is cross-sectional. Although we were able to establish an inverse correlation between the presence of vascular markings in the proximal tibia and the presence and severity of osteoarthritis, we cannot comment on cause and effect. We do not know if OA worsened, improved, or remained the same as this is a cross-sectional study rather than a longitudinal study. We are unable to say whether the increasing K-L grade is a cause of or is an effect of vascular mark changes or vice versa. The study is based on an unselected series of patients referred to the Knee Clinic in a University Teaching Hospital. We do not claim that they represent a typical adult population. We do not have clinical diagnoses or pain or function scores on these patients and we accept that there is often a difference between the radiological findings of knee osteoarthritis and a patient’s symptoms. The mean interval between X-ray and MRI scans was 3.65 months (SD 3.21, range 0.03–11.87). We did not have sequential scans to allow an assessment of vascular marks changing with time but hope to study that progression in future. A further limitation is our inability at this stage to prove that the MRI marks are blood vessels. However, a literature search failed to identify any other description of these marks on MRI scans and radiological opinion was that they fulfil the MRI criteria for veins or vessels.

## Conclusion

In conclusion, our study is the first to describe subchondral vascular marks on MRI scans and to identify an inverse relationship between those marks and the presence and distribution of knee osteoarthritis. Although cause and effect are not established, there is clearly an association. The loss of marks on MRI may be a means of diagnosing or predicting the development of osteoarthritis more accurately than by plain X-ray alone. Future work will involve a review of sequential pairs of X-rays and MRI scans and a histological analysis of the subchondral plane for vessels.

## Abbreviations

K-L: Kellgren-Lawrence
MRI: Magnetic resonance imaging
OA: Osteoarthritis
PD_SPAIR: Proton Density Spectral Presaturation with Attenuated Inversion Recovery

## References

[1] Hunter DJ, Schofield D, Callander E (2014). The individual and socioeconomic impact of osteoarthritis. Nat Rev Rheumatol.

[2] Abramson Steven B, Attur Mukundan (2009). Developments in the scientific understanding of osteoarthritis. Arthritis Research & Therapy.

[3] Trueta J, Harrison MHM (1953). The normal vascular anatomy of the femoral head in adult man. Journal of Bone and Joint Surgery-British Volume.

[4] Brookes M, Revell WJ (1998). Blood supply of bone: scientific aspects.

[5] Gray H, Standring S, Ellis H, Berkovitz BKB (2005). Gray's anatomy : the anatomical basis of clinical. Practice.

[6] Crock HV (1962). Arterial supply and venous drainage of bones of human knee joint. Anat Rec.

[7] Crock HV, Yoshizawa H (1976). Blood-supply of lumbar vertebral column. Clin Orthop Relat Res.

[8] Denham RA (1959). Hip mechanics. The Journal of bone and joint surgery British volume.

[9] Beverly M, Pflug J, Mathie R (1987). Bone - a flexible perfused sponge. J Bone Joint Surg Br..

[10] Beverly M, Murray D (2018). Factors affecting intraosseous pressure measurement. J Orthop Surg Res.

[11] Azuma H (1964). Intraosseous pressure as a measure of hemodynamic changes in bone marrow. Angiology.

[12] Ficat RP (1985). Idiopathic bone necrosis of the femoral head. Early diagnosis and treatment. The Journal of bone and joint surgery British volume.

[13] Laroche M, Ludot I, Thiechart M, Arlet J, Pieraggi M, Chiron P, Moulinier L, Cantagrel A, Puget J, Utheza G (1995). Study of the intraosseous vessels of the femoral head in patients with fractures of the femoral neck or osteoarthritis of the hip. Osteoporosis international : a journal established as result of cooperation between the European Foundation for Osteoporosis and the National Osteoporosis Foundation of the USA.

[14] Lemperg RK, Arnoldi CC (1978). The significance of intraosseous pressure in normal and diseased states with special reference to the intraosseous engorgement-pain syndrome. Clin Orthop Relat Res.

[15] Beverly M, Urban J, Murray D (2016). Factors affecting physiology of intraosseous pressure measurement. Osteoarthr Cartil.

[16] Kellgren JH, Lawrence JS (1957). Radiological assessment of osteo-arthrosis. Ann Rheum Dis.

[17] Beverly M. Bone compression, intraosseous pressure and vascular drainage. Scand J Rheumatol. 1986;(Suppl 60):24.

[18] Demondion X, Delfaut EM, Drizenko A, Boutry N, Francke JP, Cotten A (2000). Radio-anatomic demonstration of the vertebral lumbar venous plexuses: an MRI experimental study. Surgical and radiologic anatomy : SRA.

[19] Kwee RM, Kavanagh EC, Adriaensen ME (2013). Intraosseous venous drainage of pretibial varices. Skelet Radiol.

[20] Lynch JA, Roemer FW, Nevitt MC, Felson DT, Niu J, Eaton CB, Guermazi A (2010). Comparison of BLOKS and WORMS scoring systems part I. Cross sectional comparison of methods to assess cartilage morphology, meniscal damage and bone marrow lesions on knee MRI: data from the osteoarthritis initiative. Osteoarthritis and cartilage / OARS, Osteoarthritis Research Society.

[21] Majumdar S (2010). Advances in MRI of the knee for osteoarthritis.

